# Effects of one-year supplementation with *Phyllanthus niruri* on fibrosis score and metabolic markers in patients with non-alcoholic fatty liver disease: A randomized, double-blind, placebo-controlled trial

**DOI:** 10.1016/j.heliyon.2023.e16652

**Published:** 2023-05-30

**Authors:** Muhammad Radzi Abu Hassan, Rosaida Hj Md Said, Zalwani Zainuddin, Haniza Omar, Siti Maisarah Md Ali, Siti Aishah Aris, Huan-Keat Chan

**Affiliations:** a Clinical Research Centre, Hospital Sultanah Bahiyah, Jalan Langgar, 05460, Alor Setar, Kedah, Malaysia; b Medical Department, Hospital Ampang, Jalan Mewah Utara, Taman Pandan Mewah, 68000, Ampang Jaya, Selangor, Malaysia; c Medical Department, Hospital Sultanah Bahiyah, Jalan Langgar, 05460, Alor Setar, Kedah, Malaysia; d Medical Department, Hospital Selayang, Jalan Lingkaran Tengah 2, 68100 Batu Caves, Selangor, Malaysia

**Keywords:** Herbal medicine, Fibrosis, Liver cirrhosis, Non-alcoholic fatty liver disease, *Phyllanthus*

## Abstract

**Background and purpose:**

and purpose: Non-alcoholic fatty liver disease (NAFLD) is a significant global health concern with limited pharmacotherapy options. This study aimed to evaluate the effectiveness of a standardized extract of *Phyllanthus niruri* in mild-to-moderate NAFLD.

**Materials and methods:**

This was a 12-month randomized controlled trial, in which adults with a controlled attenuation parameter (CAP) score >250 dB/m and a fibrosis score <10 kPa were randomly assigned to receive a standardized *P. niruri* extract at a dose of 3,000 mg daily (n = 112) or a placebo (n = 114). The primary outcomes were changes in CAP score and liver enzyme levels, while the secondary outcomes were changes in other metabolic parameters. The analysis was performed on an intention-to-treat basis.

**Results:**

After 12 months, there was no significant difference in the change of CAP score between the intervention and control groups (−15.05 ± 36.76 dB/m vs. −14.74 ± 41.08 dB/m; p = 0.869). There was also no significant difference in the changes of liver enzyme levels between the two groups. However, the intervention group showed a significant reduction in fibrosis score, which was not observed in the control group (−0.64 ± 1.66 kPa versus 0.10 ± 1.61 kPa; p = 0.001). No major adverse events were reported in either group.

**Conclusion:**

This study showed that *P. niruri* did not significantly reduce CAP score and liver enzyme levels in patients with mild-to-moderate NAFLD. However, a significant improvement in fibrosis score was observed. Further research is needed to determine its clinical benefits at different dosages for NAFLD treatment.

## 1 Introduction

Non-alcoholic fatty liver disease (NAFLD) represents a spectrum of hepatic disorders characterized by steatosis, without competing etiologies such as alcohol consumption, chronic drug use and viral infections [1]. Approximately one-quarter of the global population are living with NAFLD [2], making it a significant public health concern. The prevalence of NAFLD has risen in tandem with obesity and diabetes, given its strong association with insulin resistance and adipose tissue dysfunction [3]. Non-alcoholic steatohepatitis (NASH) is the progressive form of NAFLD, which can eventually lead to cirrhosis and hepatocellular carcinoma [1]. Physical exercise and weight reduction are currently the mainstays of NAFLD treatment [4], as the evidence to support pharmacotherapy, including vitamin E and thiazolidinediones, is limited [5]. At present, there is no single agent approved for NAFLD treatment [6].

*Phyllanthus niruri* is a perennial herb traditionally used for a wide range of diseases, including dyspepsia, diarrhea, jaundice, kidney stones and genitourinary infections [7]. It is particularly renowned in Malay, Chinese and Ayurvedic medicine for its use in liver and kidney ailments [7,8]. Over the years, preclinical studies have shown that *P. niruri* possesses hepatoprotective, antiviral, antioxidant and anti-inflammatory activities [[9], [10], [11], [12], [13]]. Flavonoids and polyphenols are among the phytochemicals found in *P. niruri*, which exert antioxidant and hepatoprotective effects [7]. Extracts from *P. niruri* have been shown to stimulate repair mechanisms and normalize enzyme levels in liver tissues following exposure to oxidative stress caused by hepatotoxic agents, such as carbon tetrachloride, acetaminophen and nimesulide [9,14,15]. Additionally, a 50% methanolic extract of *P. niruri* with high levels of ellagic acid and phyllanthin was found to exhibit inhibitory effects against the progression of NAFLD in Sprague-Dawley rats with high-fat-diet-induced steatosis [16].

Nevertheless, clinical research into therapeutic values of *P. niruri* in liver diseases is currently limited. A randomized controlled trial comparing *P. niruri* and placebo in hepatitis B was terminated prematurely due to the absence of apparent treatment benefits [17]. In contrast, another randomized controlled trial demonstrated that a 4-week consumption of *P. niruri* significantly improved antioxidant levels and appetite in patients with alcoholic hepatitis [18]. A pilot clinical study also suggested that a long-term consumption of *P. niruri* was safe, even though its effectiveness against NAFLD was not fully demonstrated due to a limited sample size and clinical heterogeneity in patients [19]. Given the lack of evidence regarding pharmacotherapy for NAFLD, this study aimed to evaluate the effectiveness of a one-year supplementation with a formula containing a standardized extract of *P. niruri* in patients with mild-to-moderate stages of the disease.

## 2 Materials and methods

### 2.1 Study design and ethics

This study was a randomized, double-blind, placebo-controlled trial conducted in three hospitals with gastroenterology services in Malaysia. The study was registered with the National Medical Research Register under the protocol code NMRR-18-117-39930. It was also approved by the Medical Research and Ethics Committee of the Ministry of Health with the approval number 21/KKM/NIHSEC/P18-288. All participants provided written informed consent prior to enrollment.

### 2.2 Study drugs

The intervention group received Hepar-P, a commercially available liver tonic in Malaysia manufactured by Nova Laboratories. Each capsule of Hepar-P contained 250 mg of *P. niruri* extract EPN 797, which included 4% corilagin and 18% flavonoids. The control group received an equivalent number of placebo capsules containing lactose and corn starch. The *P. niruri* and placebo capsules were identical in appearance, color, smell and taste.

The daily dose of *P. niruri* used in this study was 3,000 mg (12 capsules), administered in two divided doses over a period of 12 months. The dose selection was based on several factors. Firstly, the absence of acute toxicity in Sprague-Dawley rats consuming *P. niruri* suggested its safety for consumption at high doses up to 5,000 mg/kg per day [20]. Secondly, inhibitory effects of *P. niruri* against NAFLD progression were demonstrated in Sprague-Dawley rats at a dose of 250 mg/kg (equivalent to a human dose of 40.5 mg/kg) per day [16,21]. Lasty, improvements in steatosis and transaminase levels were observed in NAFLD patients receiving treatment with rosiglitazone for one year [22].

### 2.3 Study population

Participants were required to meet the following eligibility criteria: (i) be 18 years of age or older; (ii) have a controlled attenuation parameter (CAP) score >250 dB/m (S2–S3) and a fibrosis score <10 kPa (F0–F2); (iii) have mildly to moderately raised transaminase levels (1–5 times the upper limits of normal) and an aspartate aminotransaminase (AST) to alanine aminotransaminase (ALT) ratio <0.8; and (iv) consume alcohol <20 g per day for women and <30 g per day for men. Participants were excluded if they had consumed any *Phyllanthus*-containing products within 90 days prior to recruitment or had a history of drug-induced liver injury, viral hepatitis, autoimmune liver disease, hemochromatosis, celiac disease or Wilson's disease.

### 2.4 Randomization and blinding

Study participants were randomly allocated to either the intervention or control group in a 1:1 ratio using a computer-generated randomization list. To ensure blinding, study participants, investigators and research assistants were all kept unaware of group assignments. Allocation concealment was ensured by dispensing *P. niruri* and placebo capsules in sequentially numbered containers according to the randomization list.

### 2.5 Study procedures

The recruitment process took place at the gastroenterology clinic between July and November 2018. Potential participants were informed about the study protocol and screened for eligibility. Demographic characteristics and medical history were collected at baseline, while clinical and laboratory data were gathered at baseline and during follow-up visits in months 3, 6, 9 and 12. Along with pharmacological treatment, study participants were advised to follow a set of general recommendations on weight management [23]. These recommendations included utilizing body mass index (BMI) as a tool to manage weight and adopting a healthy lifestyle by maintaining a balanced diet and engaging in regular physical activity. A diary was also used to record drug administration and any adverse events (AEs). In addition to conducting pill counts to monitor the number of capsules remaining in bottles, study participants were also contacted by phone 45 days after each visit to confirm their adherence to the treatment regimen and reinforce weight management recommendations.

During each visit, trained operators used transient elastography (FibroScan 502 Touch) to measure CAP and fibrosis scores. Additionally, trained research assistants used a Seca scale to measure weight, a Seca stadiometer to measure height and a flexible tape to measure waist circumference. Nurses collected 15 mL of blood from each participant during each visit, and all blood samples were analyzed at a central laboratory. The laboratory tests were conducted using the Cobas 8000 analyzer (Roche), except for the measurement of HbA1C which was performed using the Cobas C513 analyzer (Roche) through an immunoturbidimetric method. AST and ALT were analyzed through a photometric method, while enzymatic methods were used for alkaline phosphatase (ALP), gamma-glutamyl transferase (GGT), fasting blood glucose, total cholesterol, low-density lipoprotein (LDL) and high-density lipoprotein (HDL).

### 2.6 Outcomes

In alignment with previous investigations into NAFLD [[24], [25], [26], [27], [28]], the effectiveness of *P. niruri* in changing liver, lipid, glycemic and anthropometric profiles was evaluated. The primary outcomes were changes in CAP score, AST, ALT and AST/ALT ratio after 12 months, while the secondary outcomes were changes in fibrosis score, ALP, GGT, fasting blood glucose, HbA1C, total cholesterol, LDL, HDL, BMI and waist circumference after 12 months.

### 2.7 Statistical analysis

This study required a sample size of 90 in each group to detect a clinically meaningful difference of 15 dB/m in CAP score, based on previous studies of NAFLD treatment [[24], [25], [26], [27], [28]]. The calculation used a power of 0.80, an alpha of 0.05 (one-sided), a superiority margin of 0.10, and a population standard deviation of 40 dB/m. To account for potential dropout, the sample size was increased by 25%.

Data were analyzed using the SAS Enterprise Guide 7.1 software, with the statistical significance set at p < 0.05. Missing post-baseline data were imputed using the last-observation-carried-forward method. The intention-to-treat (ITT) analysis included all participants who received at least one dose of *P. niruri* or placebo and had at least one post-baseline efficacy assessment. All participants were included in the safety evaluation. Continuous variables were summarized using means and standard deviations (SDs), and categorical variables were summarized using frequencies and percentages. Demographic and baseline characteristics of the two groups were compared using independent t-, Pearson's Chi-square or Fisher's exact test. To analyze within- and between-group mean changes for primary and secondary outcomes, a repeated measures analysis of covariance (ANCOVA) was employed, with study site, gender, and age as covariates. The analysis encompassed data from baseline, month 3, month 6, month 9, and month 12.

## 3 Results

**Fig. 1:**
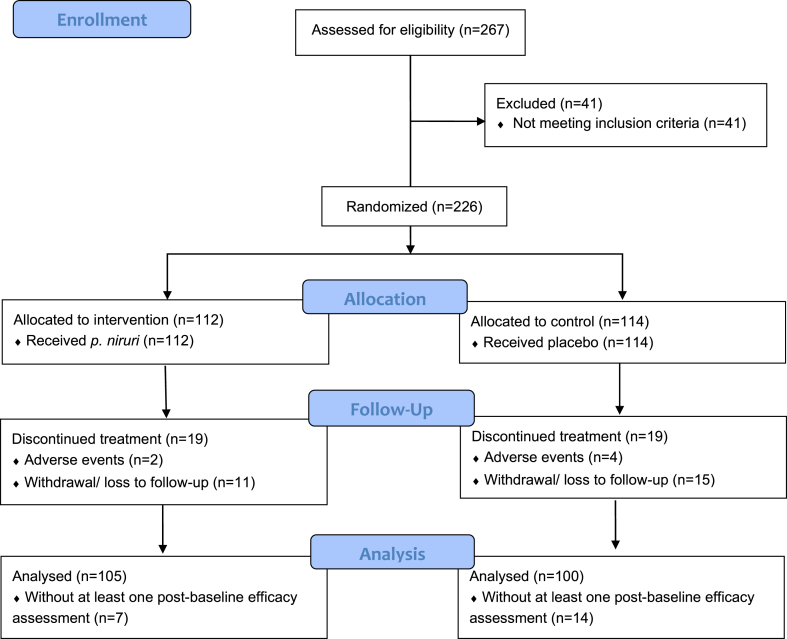
Study flow diagram.

A total of 226 eligible patients were enrolled in this study (Fig. 1). The last follow-up visit for the final enrolled participant occurred in October 2019. All participants took at least one dose of *p. niruri* (n = 112) or placebo (n = 114), but 38 of them did not complete the full treatment regimen (19 in each group). Twenty-one participants who did not undergo at least one efficacy assessment after the baseline were excluded from the efficacy analysis.

### 3.1 Characteristics of participants

The demographic and baseline characteristics of the two groups were comparable (Table 1). The mean (SD) age of participants in the intervention and control groups was 50.71 (12.26) and 49.80 (11.93) years, respectively. In both groups, over 60% of the participants were aware of their NAFLD status before the study. The most common comorbidity among the participants was hypertension.

**Table 1:** Characteristics of study participants.

Characteristics^a^	Intervention (n = 112)	Control (n = 114)
**Age in years, mean (SD)**	50.71 (12.26)	49.80 (11.93)
Gender, n (%)		
Male	56 (50.0)	51 (44.7)
Female	56 (50.0)	63 (55.3)
**Ethnicity, n (%)**		
Malay	54 (48.2)	64 (56.1)
Chinese	57 (50.9)	47 (41.2)
Indian	1 (0.9)	3 (2.6)
**Known NAFLD before the study, n (%)**	68 (60.7)	75 (65.8)
**Comorbidities, n (%)**		
Hypertension	39 (34.8)	36 (31.6)
Dyslipidemia	19 (17.0)	28 (24.6)
Diabetes mellitus	20 (17.9)	16 (14.0)
**Concomitant medications, n (%)**		
Statins	40 (35.7)	46 (40.4)
Calcium channel blockers	25 (22.3)	25 (21.9)
Metformin	29 (25.9)	16 (14.0)
ACE inhibitors	17 (15.2)	17 (14.9)
**Incomplete treatment, n (%)**	19 (17.0)	19 (16.7)
Due to AE**s**	2 (1.8)	4 (3.5)
Due to withdrawal/loss to follow-up	17 (15.2)	15 (13.2)

ACE, angiotensin-converting enzyme; AE, adverse event; n, number of participants; NAFLD, non-alcoholic fatty liver disease; SD, standard deviation.

a The two groups did not significantly differ in their characteristics.

### 3.2 Effectiveness

**Fig. 2:**
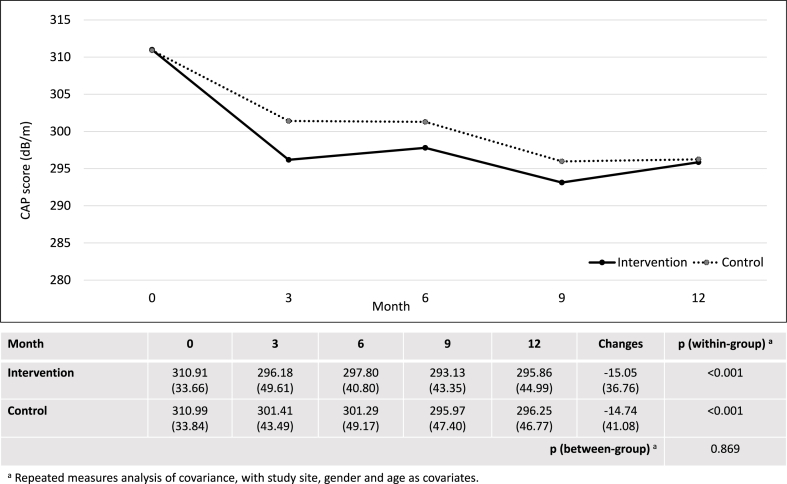
Changes in controlled attenuation parameter score (dB/m).

**Fig. 3:**
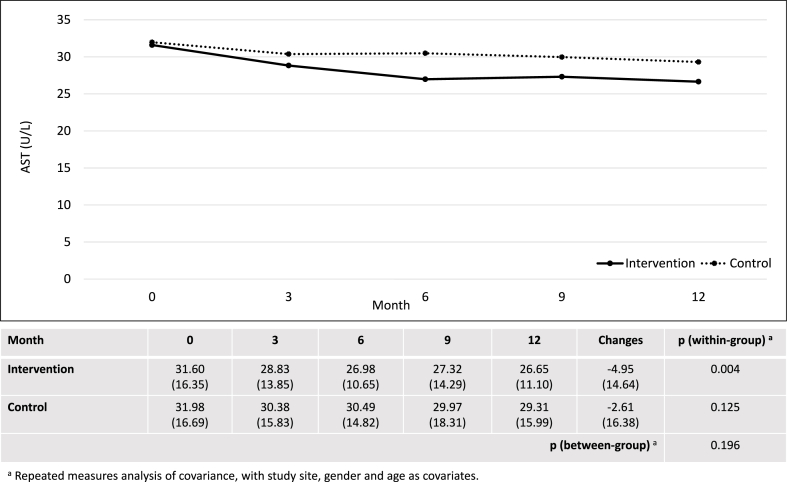
Changes in aspartate aminotransaminase (U/L).

**Fig. 4:**
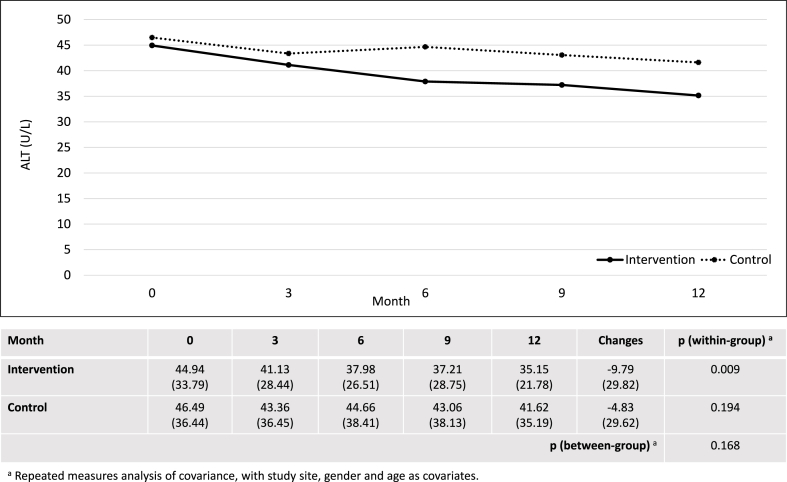
Changes in alanine aminotransaminase (U/L).

**Fig. 5:**
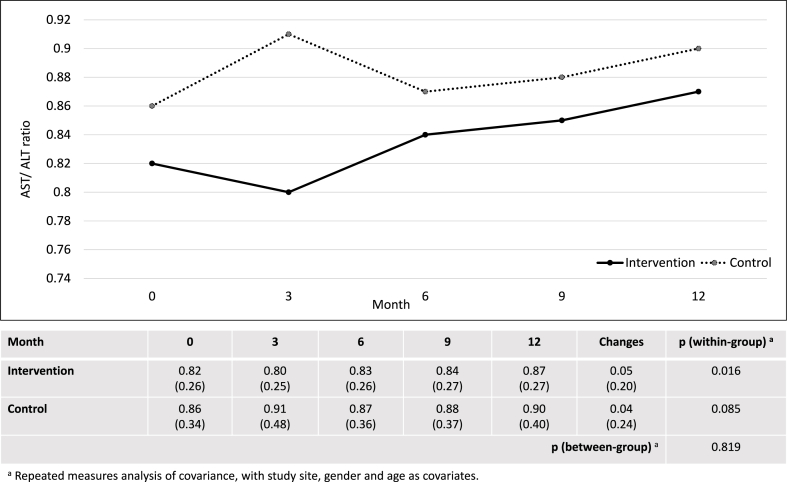
Changes in aspartate aminotransaminase to alanine aminotransaminase ratio.

After 12 months, the intervention group showed significant within-group changes in CAP score, AST, ALT, AST/ALT ratio, fibrosis score, total cholesterol and HDL. However, there were no significant differences between the intervention and control groups in changes of CAP score (−15.05 ± 36.76 dB/m vs. −14.74 ± 41.08 dB/m; p = 0.869), AST (−4.95 ± 14.64 U/L vs. −2.61 ± 16.38 U/L; p = 0.196), ALT (−9.79 ± 29.82 U/L vs. −4.83 ± 29.62 U/L; p = 0.168) and AST/ALT ratio (0.05 ± 0.20 versus 0.04 ± 0.24; p = 0.819) (Fig. 2, Fig. 3, Fig. 4, Fig. 5). Although there was a decrease in ALP, GGT, LDL, and waist circumference in the intervention group, these changes were not statistically significant (Table 2). Nonetheless, the intervention group showed a significant reduction in fibrosis score, which was not observed in the control group (−0.64 ± 1.66 kPa versus 0.10 ± 1.61 kPa; p = 0.001).

**Table 2:** Changes in secondary outcomes.

Outcomes	Intervention (n = 105)					Control (n = 100)					p (between-group)^a^
	Mean	(SD)	Mean Changes	(SD)	p (within-group)^a^	Mean	(SD)	Mean Changes	(SD)	p (within-group)^a^	
**Fibrosis score (kPa)**			−0.64	(1.66)	0.004			0.10	(1.61)	0.615	0.001
Baseline	6.21	(1.61)				5.81	(1.73)				
3 months	5.75	(1.82)				5.89	(2.29)				
6 months	5.66	(2.49)				5.78	(1.88)				
9 months	5.56	(1.71)				5.55	(1,96)				
12 months	5.57	(1.77)				5.91	(2.10)				
**ALP (U/L)**			−0.06	(12.60)	0.967			−2.23	(13.36)	0.193	0.224
Baseline	82.46	(22.76)				80.72	(24.08)				
3 months	82.61	(23.75)				79.51	(22.95)				
6 months	82.69	(23.01)				79.66	(24.90)				
9 months	82.46	(23.51)				77.03	(21.79)				
12 months	82.39	(23.90)				78.43	(22.44)				
**GGT (U/L)**			−4.49	(28.30)	0.195			−2.36	(27.66)	0.468	0.506
Baseline	50.29	(39.31)				49.37	(41.35)				
3 months	54.67	(49.83)				44.05	(29.68)				
6 months	47.34	(39.99)				46.03	(36.90)				
9 months	43.97	(41.62)				42.50	(32.07)				
12 months	45.80	(45.00)				47.04	(46.48)				
**HbA1C (%)**			0.11	(0.85)	0.195			0.07	(0.46)	0.175	0.529
Baseline	5.94	(0.77)				5.95	(0.71)				
3 months	5.88	(0.76)				5.89	(0.79)				
6 months	5.99	(0.85)				5.96	(0.73)				
9 months	5.96	(0.89)				5.95	(0.70)				
12 months	6.05	(0.94)				6.01	(0.75)				
**FBG (mmol/L)**			0.40	(2.45)	0.130			0.15	(1.20)	0.199	0.395
Baseline	5.42	(1.16)				5.41	(1.52)				
3 months	5.46	(1.13)				5.40	(1.31)				
6 months	5.48	(1.37)				5.33	(1.33)				
9 months	5.46	(1.48)				5.45	(1.67)				
12 months	5.83	(2.80)				5.57	(1.69)				
**TC (mmol/L)**			−0.17	(0.83)	0.033			−0.09	(0.83)	0.395	0.590
Baseline	5.29	(1.15)				5.35	(0.94)				
3 months	5.17	(1.02)				5.32	(0.97)				
6 months	5.17	(1.04)				5.39	(0.97)				
9 months	5.06	(1.08)				5.28	(1.00)				
12 months	5.12	(1.02)				5.28	(1.04)				
**HDL (mmol/L)**			−0.06	(0.21)	0.013			−0.01	(0.18)	0.584	0.082
Baseline	1.30	(0.34)				1.33	(0.29)				
3 months	1.25	(0.29)				1.33	(0.32)				
6 months	1.24	(0.32)				1.32	(0.32)				
9 months	1.25	(0.34)				1.33	(0.32)				
12 months	1.24	(0.32)				1.32	(0.30)				
**LDL (mmol/L)**			−0.08	(0.71)	0.395			−0.09	(0.71)	0.283	0.721
Baseline	3.14	(1.01)				3.25	(0.83)				
3 months	3.24	(0.80)				3.24	(0.80)				
6 months	3.33	(0.87)				3.33	(0.87)				
9 months	3.20	(0.82)				3.20	(0.82)				
12 months	3.06	(1.01)				3.17	(0.93)				
**BMI (kg/m2)**			0.04	(1.21)	0.815			−0.01	(0.99)	0.916	0.834
Baseline	29.22	(4.17)				28.54	(4.99)				
3 months	29.40	(4.36)				28.58	(5.15)				
6 months	29.36	(4.54)				28.61	(5.34)				
9 months	29.40	(4.64)				28.53	(5.37)				
12 months	29.26	(4.47)				28.53	(5.22)				
**WC (cm)**			−0.28	(5.32)	0.599			−0.52	(5.50)	0.412	0.608
Baseline	96.05	(9.32)				94.65	(11.64)				
3 months	95.84	(9.99)				94.07	(10.87)				
6 months	95.99	(9.43)				93.68	(11.52)				
9 months	96.86	(12.51)				93.49	(10.98)				
12 months	95.77	(9.92)				94.13	(12.02)				

ALP, alkaline phosphatase; BMI, body mass index; EOT, FBG, fasting blood glucose; GGT, glutamyl transferase; HbA1C, hemoglobin A1C; HDL, high-density lipoprotein; LDL, low-density lipoprotein; n, number of participants; SD, standard deviation; TC, total cholesterol; WC, waist circumference.

a Repeated measures analysis of covariance, with study site, gender and age as covariates.

### 3.3 Safety

During the study period, a total of 88 AEs were recorded, with similar numbers observed in the two groups (Table 3). The most frequently reported AEs in the intervention group included fever, headache, diarrhea, cough, upper respiratory tract infection and gastritis. While two participants in each group experienced serious AEs, no deaths were reported.

**Table 3:** Adverse events.

Aspects	Intervention (n = 112)	Control (n = 114)
Adverse events, n	43 (38.4)	45 (39.5)
Serious adverse events, n	2 (1.8)	2 (1.8)
Common adverse events, n		
Fever	5 (4.5)	18 (15.8)
Headache	6 (5.4)	6 (5.3)
Diarrhea	4 (3.6)	5 (4.4)
Cough	3 (2.7)	4 (3.5)
Upper respiratory tract infection	3 (2.7)	4 (3.5)
Gastritis	1 (0.9)	4 (3.5)

n, number of events.

## 4 Discussion

To our knowledge, this study represents the first large-scale randomized controlled trial to investigate the effectiveness of *P. niruri* in NAFLD. A key strength of this study was its duration of one year, which allowed for a comprehensive evaluation of the long-term effects of *P. niruri* supplementation. Additionally, this study specifically targeted patients with mild-to-moderate stages of NAFLD, who are most likely to benefit from *P. niruri*. Although the standardized extract of *P. niruri* did not demonstrate superiority over placebo in alleviating steatosis, it exhibited clinical benefits by reducing the fibrosis score.

In this study, the intervention and control groups showed a similar decrease in CAP score, which does not support the hypothesis that *P. niruri* inhibits steatosis, as suggested by an animal study [16]. However, it is noteworthy that the study team adopted a conservative dosing strategy, which may account for the absence of significant difference in CAP score reduction between the groups. The dose of *P. niruri* extract used in this study was determined based on a consensus reached by the study team to adopt the lowest of the three doses tested in rats with high-fat-diet-induced steatosis [16]. The selected dose was 3,000 mg daily, which was converted from a daily dose of 250 mg/kg in rats. A higher dose was not chosen, given that another clinical trial on alcoholic steatosis used a much lower dose of *P. niruri* (1,000 mg daily) [18]. While *P. niruri* was shown to reduce visceral adipose tissue weight and improve liver histology only at a dose of 1,000 mg/kg in rats [16], the impact of a higher dose of *P. niruri* in humans remains unclear.

The improvement in steatosis observed in both the intervention and control groups in this study was likely due to changes in lifestyle [29]. All participants were instructed to follow a set of weight management recommendations [23], which included consuming a moderate amount of nutritious food and engaging in 45–60 min of regular exercise. The decrease in CAP score in both the groups was accompanied by a declining trend in total cholesterol, LDL and waist circumference. However, toward the end of the study, it was possible that the weight management recommendations were not strictly followed, resulting in a lack of reduction in BMI.

Apart from CAP score, the intervention group showed a significant within-group reduction in AST and ALT levels in this study. The findings align with a preclinical study, which reported the ability of *P. niruri* to lower liver enzyme levels at a dose as low as 250 mg/kg in rats [16]. A large-scale cohort study of more than 5000 men reported that elevated ALT is an independent predictor of NAFLD, even when it remains within the normal range [30]. Abnormal liver enzymes are also well-established determinants of NAFLD progression [31]. Although the difference in changes of liver enzyme levels between the intervention and control groups did not reach statistical significance, there was a declining trend with a larger magnitude in the intervention group, suggesting the potential *anti*-NAFLD effects of *P. niruri* in humans. Nevertheless, demonstrating significant between-group differences would require a higher dose of *P. niruri*, as seen in the case of the CAP score.

In this study, it was observed that *P. niruri* can effectively reduce fibrosis score. This is in line with mounting evidence suggesting the reversibility of liver fibrosis of different etiologies [32,33]. While there is a positive association between the grade of angiogenesis and liver fibrosis [34], a preclinical study revealed that *P. niruri* has antiangiogenic and adipocytokine-regulating activities [35]. These properties are believed to contribute to the effectiveness of *P. niruri* in suppressing the pathological expansion of adipose tissues, improving insulin signaling and preventing inflammation [16]. The current findings suggest that even at a relatively low dose, *P. niruri* can improve fibrosis, adding to the existing literature on its potential therapeutic benefits for NAFLD.

This study also demonstrated the safety of *P. niruri* when used at a dose of 3,000 mg for an extended period. These findings were consistent with a pilot clinical study of *P. niruri*, which showed no major adverse events when a smaller dose of 1,500 mg was administered daily for 48 weeks [19]. Another clinical trial evaluating *P. niruri* for hepatitis B at a dose of 250 mg twice daily also reported that it was generally well-tolerated [36].

One of the study limitations is the absence of histological examination through liver biopsy due to its invasive nature. The decision not to use liver biopsy was made based on lessons learnt from a previous pilot study [19], in which many participants refused post-treatment liver biopsy. However, transient elastography has been demonstrated to have acceptable accuracy in diagnosing and staging steatosis and fibrosis [37], leading to an increasing trend in the use of CAP and fibrosis scores as endpoints for studies of NAFLD treatment [[24], [25], [26], [27], [28]]. Another limitation of this study is that the practice of maintaining a balanced diet and engaging in regular exercise was not specifically evaluated. Nonetheless, participants were reminded of the weight management recommendations after each follow-up visit throughout the study period. Additionally, while insulin resistance is a well-established factor in the development of NAFLD, the potential of *P. niruri* in mitigating insulin resistance was not explored in this study. Moreover, this study did not investigate the effects of different dosing strategies for *P. niruri*. The study duration was also likely not long enough to assess the potential of *P. niruri* in preventing full-blown cirrhosis. Therefore, further investigation into the use of *P. niruri* at higher doses and for longer treatment durations is warranted.

## 5 Conclusion

Although one-year supplementation with *P. niruri* was not shown to be more effective than placebo in reducing CAP score and liver enzyme levels in patients with mild-to-moderate NAFLD, it demonstrated clinical benefits by reducing fibrosis score. The long-term consumption of *P. niruri* was also found to be safe. Future research could focus on exploring different dosing strategies of *P. niruri* and its effectiveness in mitigating insulin resistance.

## Author contribution statement

M. R. Abu Hassan, H. K. Chan: Conceived and designed the experiments; Analyzed and interpreted the data; Contributed reagents, materials, analysis tools or data; Wrote the paper.

R. Hj Md Said, Z. Zainuddin, H. Omar, S. M. Md Ali, S. A. Aris: Conceived and designed the experiments; Performed the experiments; Wrote the paper.

## Data availability statement

Data will be made available on request.

## Funding

This study was supported by the grant provided by the National Key Economic Area Entry Point Project 1 of the Economic Transformation Program in Malaysia. Nova Laboratories provided both the study product (Hepar-P) and placebo used in the study.

## Declaration of competing interest

The authors declare the following financial interests/personal relationships which may be considered as potential competing interests: Huan-Keat Chan reports equipment, drugs, or supplies was provided by Nova Laboratories Ltd.

## References

[1] Benedict M., Zhang X. (2017). Non-alcoholic fatty liver disease: an expanded review. World J. Hepatol..

[2] Younossi Z.M., Koenig A.B., Abdelatif D., Fazel Y., Henry L., Wymer M. (2016). Global epidemiology of nonalcoholic fatty liver disease-Meta-analytic assessment of prevalence, incidence, and outcomes. Hepatology.

[3] Godoy-Matos A.F., Silva Júnior W.S., Valerio C.M. (2020). NAFLD as a continuum: from obesity to metabolic syndrome and diabetes. Diabetol. Metab. Syndrome.

[4] Moore M.P., Cunningham R.P., Dashek R.J., Mucinski J.M., Rector R.S. (2020). A fad too far? Dietary strategies for the prevention and treatment of NAFLD. Obesity.

[5] Leoni S., Tovoli F., Napoli L., Serio I., Ferri S., Bolondi L. (2018). Current guidelines for the management of non-alcoholic fatty liver disease: a systematic review with comparative analysis. World J. Gastroenterol..

[6] Attia S.L., Softic S., Mouzaki M. (2021). Evolving role for pharmacotherapy in NAFLD/NASH. Clin. Transl. Sci..

[7] Lee N.Y., Khoo W.K., Adnan M.A., Mahalingam T.P., Fernandez A.R., Jeevaratnam K. (2016). The pharmacological potential of Phyllanthus niruri. J. Pharm. Pharmacol..

[8] Ezzat M.I., Okba M.M., Ahmed S.H., El-Banna H.A., Prince A., Mohamed S.O. (2020). In-depth hepatoprotective mechanistic study of Phyllanthus niruri: in vitro and in vivo studies and its chemical characterization. PLoS One.

[9] Bhattacharjee R., Sil P.C. (2007). Protein isolate from the herb, Phyllanthus niruri L. (Euphorbiaceae), plays hepatoprotective role against carbon tetrachloride induced liver damage via its antioxidant properties. Food Chem. Toxicol..

[10] Manjrekar A.P., Jisha V., Bag P.P., Adhikary B., Pai M.M., Hegde A. (2008). Effect of Phyllanthus niruri Linn. treatment on liver, kidney and testes in CCl4 induced hepatotoxic rats. Indian J. Exp. Biol..

[11] Venkateswaran P.S., Millman I., Blumberg B.S. (1987). Effects of an extract from Phyllanthus niruri on hepatitis B and woodchuck hepatitis viruses: in vitro and in vivo studies. Proc. Natl. Acad. Sci. U.S.A..

[12] Bagalkotkar G., Sagineedu S.R., Saad M.S., Stanslas J. (2006). Phytochemicals from Phyllanthus niruri Linn. and their pharmacological properties: a review. J. Pharm. Pharmacol..

[13] Obidike I.C., Salawu O.A., Ndukuba M., Okoli C.O., Osunkwo U.A. (2010). The anti-inflammatory and antinociceptive properties of the chloroform fraction from Phyllanthus niruri plant is mediated via the peripheral nervous system. J. Diet. Suppl..

[14] Chatterjee M., Sil P.C. (2006). Hepatoprotective effect of aqueous extract of Phyllanthus niruri on nimesulide-induced oxidative stress in vivo. Indian J. Biochem. Biophys..

[15] Bhattacharjee R., Sil P.C. (2006). The protein fraction of Phyllanthus niruri plays a protective role against acetaminophen induced hepatic disorder via its antioxidant properties. Phytother Res..

[16] Al Zarzour R.H., Ahmad M., Asmawi M.Z., Kaur G., Saeed M.A.A., Al-Mansoub M.A. (2017). Phyllanthus niruri standardized extract alleviates the progression of non-alcoholic fatty liver disease and decreases atherosclerotic risk in Sprague-Dawley rats. Nutrients.

[17] Baiguera C., Boschetti A., Raffetti E., Zanini B., Puoti M., Donato F. (2018). Phyllanthus niruri versus placebo for chronic hepatitis B virus infection: a randomized controlled trial. Complement. Med. Res..

[18] Sowjanya K., Girish C., Bammigatti C., Prasanna Lakshmi N.C. (2021). Efficacy of Phyllanthus niruri on improving liver functions in patients with alcoholic hepatitis: a double-blind randomized controlled trial. Indian J. Pharmacol..

[19] Hassan M.R.A., Mustapha N.R.N., Jaya F., Arjunan S., Ooi E.T., Said R.M. (2017). Efficacy and safety of Phyllanthus niruri in non-alcoholic steatohepatitis treatment: pilot study from Malaysia. J. Pharm. Pract. Commun. Med..

[20] Asare G.A., Addo P., Bugyei K., Gyan B., Adjei S., Otu-Nyarko L.S. (2011). Acute toxicity studies of aqueous leaf extract of Phyllanthus niruri. Interdiscipl. Toxicol..

[21] Shin J.W., Seol I.C., Son C.G. (2010). Interpretation of animal dose and human equivalent dose for drug development. J. Korean Med..

[22] Ratziu V., Giral P., Jacqueminet S., Charlotte F., Hartemann-Heurtier A., Serfaty L. (2008). Rosiglitazone for nonalcoholic steatohepatitis: one-year results of the randomized placebo-controlled fatty liver improvement with rosiglitazone therapy (FLIRT) trial. Gastroenterology.

[23] Ministry of Health M Weight Management 2012. http://www.myhealth.gov.my/en/weight-management/.

[24] Papapostoli I., Lammert F., Stokes C.S. (2016). Effect of short-term vitamin D correction on hepatic steatosis as quantified by controlled attenuation parameter (CAP). J. Gastrointest. Liver Dis..

[25] Honda Y., Kessoku T., Sumida Y., Kobayashi T., Kato T., Ogawa Y. (2017). Efficacy of glutathione for the treatment of nonalcoholic fatty liver disease: an open-label, single-arm, multicenter, pilot study. BMC Gastroenterol..

[26] Lee Y.H., Kim J.H., Kim S.R., Jin H.Y., Rhee E.J., Cho Y.M. (2017). Lobeglitazone, a novel thiazolidinedione, improves non-alcoholic fatty liver disease in type 2 diabetes: its efficacy and predictive factors related to responsiveness. J. Kor. Med. Sci..

[27] Rahimlou M., Yari Z., Hekmatdoost A., Alavian S.M., Keshavarz S.A. (2016). Ginger supplementation in nonalcoholic fatty liver disease: a randomized, double-blind, placebo-controlled pilot study. Hepat. Mon..

[28] Teufelhart M., Hofer H., Haslacher H., Winker R., Meyer B., Ferenci P. (2016).

[29] Franco I., Bianco A., Mirizzi A., Campanella A., Bonfiglio C., Sorino P. (2020). Physical activity and low glycemic index Mediterranean diet: main and modification effects on NAFLD score. Results from a randomized clinical trial. Nutrients.

[30] Chang Y., Ryu S., Sung E., Jang Y. (2007). Higher concentrations of alanine aminotransferase within the reference interval predict nonalcoholic fatty liver disease. Clin. Chem..

[31] Campos-Murguía A., Ruiz-Margáin A., González-Regueiro J.A., Macías-Rodríguez R.U. (2020). Clinical assessment and management of liver fibrosis in non-alcoholic fatty liver disease. World J. Gastroenterol..

[32] Sun M., Kisseleva T. (2015). Reversibility of liver fibrosis. Clin. Res. Hepatol. Gastroenterol..

[33] Sun Y.M., Chen S.Y., You H. (2020). Regression of liver fibrosis: evidence and challenges. Chin. Med. J..

[34] Elpek G. (2015). Angiogenesis and liver fibrosis. World J. Hepatol..

[35] Zarzour R.H.A., Alshawsh M.A., Asif M., Al-Mansoub M.A., Mohamed Z., Ahmad M. (2018). Adipocytokine regulation and antiangiogenic activity underlie the molecular mechanisms of therapeutic effects of Phyllanthus niruri against non-alcoholic fatty liver disease. Nutrients.

[36] Baiguera C., Boschetti A., Raffetti E., Zanini B., Puoti M., Donato F. (2018). Phyllanthus niruri versus placebo for chronic hepatitis B virus infection: a randomized controlled trial. Complement. Med. Res..

[37] Selvaraj E.A., Mózes F.E., Jayaswal A.N.A., Zafarmand M.H., Vali Y., Lee J.A. (2021). Diagnostic accuracy of elastography and magnetic resonance imaging in patients with NAFLD: a systematic review and meta-analysis. J. Hepatol..

